# Sex-dependent differences in behavioral and immunological responses to antibiotic and bacteriophage administration in mice

**DOI:** 10.3389/fimmu.2023.1133358

**Published:** 2023-05-25

**Authors:** Łukasz Grabowski, Karolina Pierzynowska, Katarzyna Kosznik-Kwaśnicka, Małgorzata Stasiłojć, Grażyna Jerzemowska, Alicja Węgrzyn, Grzegorz Węgrzyn, Magdalena Podlacha

**Affiliations:** ^1^ Laboratory of Bacteriophage Therapy, Institute of Biochemistry and Biophysics, Polish Academy of Sciences, Gdansk, Poland; ^2^ Department of Molecular Biology, Faculty of Biology, University of Gdansk, Gdansk, Poland; ^3^ Department of Medical Microbiology, Faculty of Medicine, Medical University of Gdansk, Gdansk, Poland; ^4^ Department of Cell Biology and Immunology, Intercollegiate Faculty of Biotechnology of University of Gdansk and Medical University of Gdansk, Gdansk, Poland; ^5^ Department of Animal and Human Physiology, Faculty of Biology, University of Gdansk, Gdansk, Poland; ^6^ Phage Therapy Center, University Center of Applied and Interdisciplinary Research, Gdansk, Poland

**Keywords:** antibiotics, bacteriophage, males and females, behavior, immune system, mice

## Abstract

**Introduction:**

The problem of antibiotic resistance is a global one, involving many industries and entailing huge financial outlays. Therefore, the search for alternative methods to combat drug-resistant bacteria has a priority status. Great potential is seen in bacteriophages which have the natural ability to kill bacterial cells. Bacteriophages also have several advantages over antibiotics. Firstly, they are considered ecologically safe (harmless to humans, plants and animals). Secondly, bacteriophages preparations are readily producible and easy to apply. However, before bacteriophages can be authorized for medical and veterinary use, they must be accurately characterized *in vitro* and *in vivo* to determinate safety.

**Methods:**

Therefore, the aim of this study was to verify for the first time the behavioral and immunological responses of both male and female mice (C57BL/6J) to bacteriophage cocktail, composed of two bacteriophages, and to two commonly used antibiotics, enrofloxacin and tetracycline. Animal behavior, the percentage of lymphocyte populations and subpopulations, cytokine concentrations, blood hematological parameters, gastrointestinal microbiome analysis and the size of internal organs, were evaluated.

**Results:**

Unexpectedly, we observed a sex-dependent, negative effect of antibiotic therapy, which not only involved the functioning of the immune system, but could also significantly impaired the activity of the central nervous system, as manifested by disruption of the behavioral pattern, especially exacerbated in females. In contrast to antibiotics, complex behavioral and immunological analyses confirmed the lack of adverse effects during the bacteriophage cocktail administration.

**Discussion:**

The mechanism of the differences between males and females in appearance of adverse effects, related to the behavioral and immune functions, in the response to antibiotic treatment remains to be elucidated. One might imagine that differences in hormones and/or different permeability of the blood-brain barrier can be important factors, however, extensive studies are required to find the real reason(s).

## 1 Introduction

While the threat of antibiotic resistance is increasing, the interest in the use of bacteriophages to treat bacterial infections, known as bacteriophage therapy, has rapidly grown, especially in the context of veterinary (1), poultry industry (1) and public health (2). There are 2.8 million infections with antibiotic-resistant bacteria in the United States each year, with a minimum of 35.000 cases resulting in death (3). It is projected that by 2050, the annual number of deaths worldwide caused by this type of infection will be at ten million people (4). It is worth nothing that at the root of the antibiotic crisis, it is not only their use in medicine, but also in the treatment of livestock. Indeed, about two-thirds of the tonnage of global antibiotic use are commonly employed to combat bacterial infections in food-animal production (5). *Salmonella enterica* is one of the most common pathogens causing gastrointestinal diseases in the European Union. In United States, based on data obtained from the Center for Disease Control and Prevention, it is estimated that this bacterium causes about 1.2 million cases of food product contamination, which translates into 23.000 hospitalizations and 450 deaths each year (3). Although the level of antibiotic resistance of different *Salmonella* serovars varies from country to country (6), the problem is global and requires the implementation of alternative methods to control the infection (7).

Bacteriophage therapy uses the natural ability of bacteriophages to kill bacterial cells. Bacteriophages have also several advantages over antibiotics, as they are considered ecologically safe (harmless to humans, plants and animals), and bacteriophage preparations are readily producible and easy to apply. The concentration of an antibiotic introduced into the human organism decreases with time (due to natural drug clearance from the body), whereas bacteriophages continue to multiply, decreasing as soon as sensitive bacterial cells are eliminated (8). However, before bacteriophages can be authorized for medical and veterinary use, they must be accurately characterized *in vitro* and then *in vivo* to determinate their safety. Despite bacteriophages being specific to their bacterial hosts, there are a growing number of reports about interactions of bacteriophages with eukaryotic cells. These impacts can, to varying degrees, involve not only tissues or organs, but even entire systems, including the immune system or central nervous system, as reviewed recently (9). While there are reports on the characterization and safety of bacteriophages tested *in vitro*, animal studies are still in the minority, and systematic comparison of effects of administration of bacteriophages and antibiotics *in vivo* is, to our knowledge, absent in the literature, especially regarding functions of the brain and the immune system.

Therefore, the aim of this study was to verify the behavioral and immunological responses to a bacteriophage cocktail, composed of two bacteriophages, and to two commonly used antibiotics, enrofloxacin and tetracycline, in female and male C57BL/67 mice. Animal behavior, the percentage of lymphocyte populations and subpopulations, cytokine concentrations, blood hematological parameters, gastrointestinal microbiome analysis and the size of internal organs, were evaluated.

## 2 Materials and methods

### 2.1 Animals

**Figure 1:**
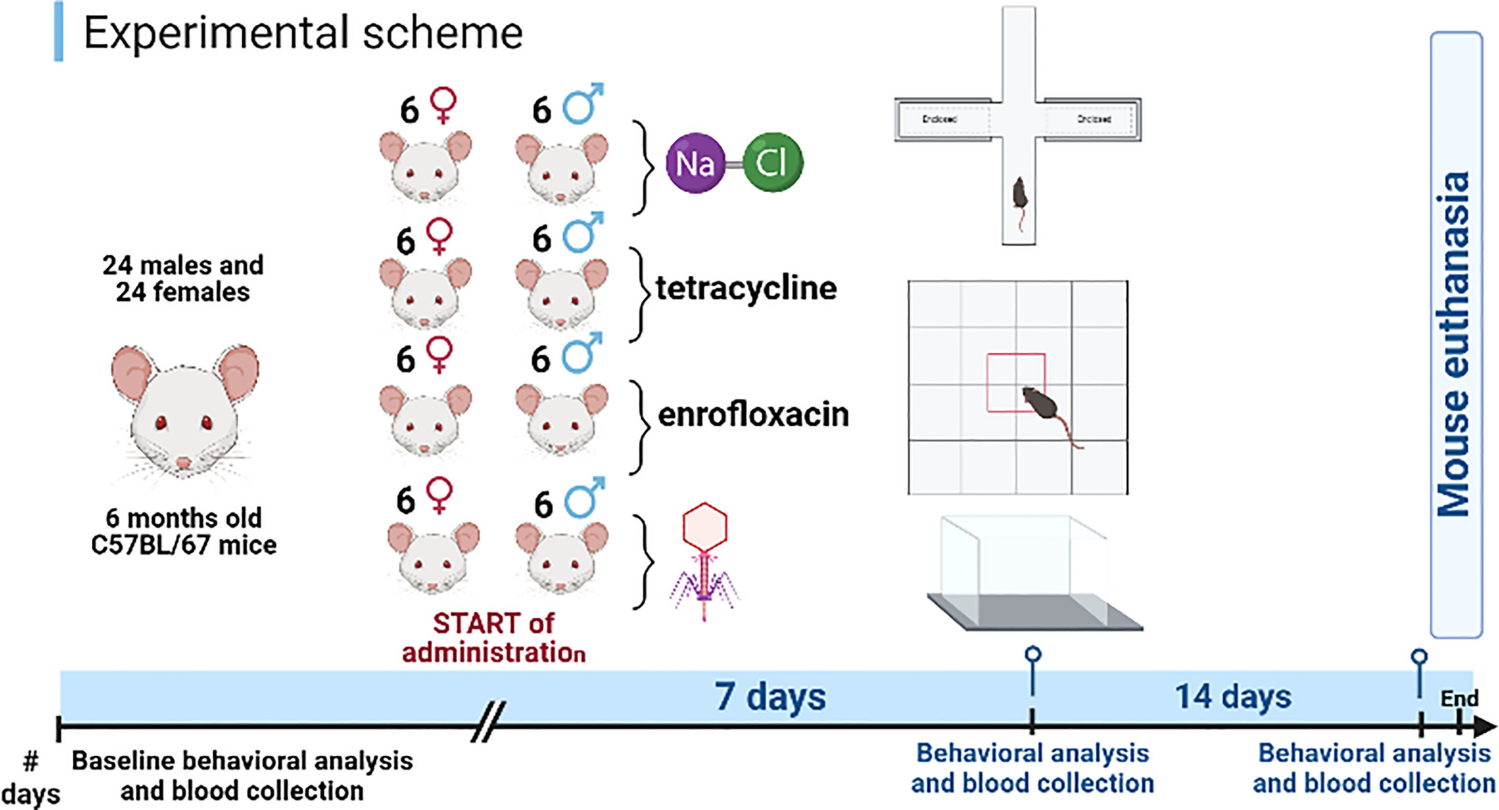
Schematic diagram of the experiment. Created using BioRender software.

The experiments were conducted with male (n=24) and female (n=24) C57BL/6J mice. At the start of the experiment, all animals were 6 months old. The experiments were performed with animals of both sexes, to assess any differences in responses to administration of different types of bacteriophages and antibiotics (**Figure 1**).

The mice were housed in a ventilated animal room (15 air changes per hour) in a laboratory setting. Stable conditions were maintained: artificial lighting (12 hours light/12 hours dark), ambient temperature (22 ± 2°C), humidity (50 ± 5%) with access to food and tap water *ad libitum*. Mice were maintained in approved laboratory cages 15 cm high and at least 400 cm^2^ in size. To ensure the most optimal enrichment of the environment, suitable attractants and accessories for rodents were used.

The animal house in which the mice were placed meets the requirements of the Law on the Protection of Animals Used for Scientific or Educational Purposes, dated on January 15, 2015 (Journal of Laws dated on February 26, 2015), as well as the recommendations of the European Commission on the welfare of animals used in scientific experiments. All experiments were approved by the Local Ethics Committee for Experimental Animals in Bydgoszcz (permission number 02/2022, dated on January 19, 2022).

### 2.2 Preparation and purification of high-titer bacteriophage lysates

Due to huge diversity of bacteriophages, we aimed to study bacteriophages representing various types of viruses, which differ in head morphology and genome size. Therefore, the following bacteriophages isolated from the environment, were employed: vB_ Sen-TO17 and vB_SenM-2. Bacteriophage vB_ Sen-TO17 is a caudate bacteriophage with a virion composed of head and tail (head diameter: 48 x 46 nm, tail length: 121 nm), bearing dsDNA as a genetic material (genome size is 41,658 bp), and infecting *Salmonella enterica* strains (10, 11). vB_SenM-2 is a *S.enterica* – specific, caudate bacteriophage (head diameter: 84 x 79 nm, tail length: 111 nm) with a dsDNA genome (the genome size is 158,986 bp) (10, 12).

The bacteriophages included in the bacteriophage cocktail were prepared according to the protocol described previously (13). Briefly, all bacteriophages were propagated in susceptible bacterial strains to obtain high titer. To avoid contamination with bacteria-derived products, including lipopolysaccharide (LPS) which reveals toxic features to eukaryotic cells, bacteriophages were concentrated with polyethylene glycol (PEG8000) (BioShop, Burlington, Ontario, Canada) and purified by ultracentrifugation at 95,000 × *g* for 2.5 hours, at 4°C (Avanti JXN-26, rotor JLA-8000, Beckman Coulter, Indianapolis, USA) in a CsCl density gradient (14). Purified bacteriophages were tested for a lack of toxic contaminants using Purified Thermo Scientific™ Pierce ™ LAL Chromogenic Endotoxin Quantitation Kit (catalog number: 12117850; Thermo Fisher Scientific Inc., Paisley, UK). To remove residual CsCl, 1 ml of bacteriophages were dialyzed against 300 ml of 3M NaCl, using a dialysis membrane (ZelluTrans, MWCO: 12.000-14.000, serial number: E674.1; Roth, Germany) for 7 days at 4°C. The NaCl was replaced every 12 hours (15).

The bacteriophages were characterized in terms of survival under various physicochemical conditions (stability in pH range between 1.8 and 12; stability in ethanol, chloroform, DMSO and acetone; stability in temperature range between -80°C and 95°C), and their effectiveness in combating various *Salmonella enterica* serovars *in vitro* was verified (10, 16).

### 2.3 Experimental groups

Experiments were conducted with eight groups for male and female mice, (i) control (receiving saline (0.9% NaCl), 0.1 ml), (ii) tetracycline, (iii) enrofloxacin, and (iv) bacteriophage cocktail. Each group consisted of six mice. The cocktail was administered at 10^9^ PFU/ml (0.1 ml), whereas enrofloxacin (Scanflox, Scanvet, Warsaw, Poland) at 5 mg/kg body weight and tetracycline (catalog number: 200-481-9; Merck, Darmstadt, Germany) at 20 mg/kg body weight, orally every day by using an oro-gastric probe, for fourteen days.

### 2.4 Locomotor activity in actometers

The locomotor activity of animals was measured using actometers (Opto Varimex Minor, Columbus, USA). The actometer consists of four plexiglass walls measuring 43 x 43 x 20 cm. At the moment of movement, a photocell is used to record each interruption of the infrared beam, which is then counted by a digital counter. The movements analyzed in this test are divided into horizontal (movements in the horizontal plane), vertical (movements in the vertical plane), and ambulatory (such as during body cleaning). The animals’ locomotor activity was recorded for 10 minutes three times, before the start of administration, after one week, and after fourteen days of administration of bacteriophage cocktail, antibiotic or saline. Measurements were taken at a fixed time, between 4 p.m. and 6 p.m., according to the method described previously (17).

### 2.5 Analysis of anxiety behavior in the open field test

The open field test allows for the assessment of the severity of the level of fear towards the stress factor of the open space, and also determines locomotor activity and the degree of exploration in a new hostile environment. Rodents are inherently prone to darkened, enclosed spaces, fear of open spaces and heights, so the test was conducted in a 100 x 100 x 60 cm box (usually made of white – colored boards), additionally exposed to a light source which intensifies the sense of fear. The floor of the box was divided into equal parts, among which central and peripheral squares were distinguished. Mice were placed in the test, always in the same position (e.g. with their head to one of the corners). The experiments were carried out at a fixed time, between 2 and 3 p.m. The locomotor activity of the exploring animal was measured by the number of squares crossed. The level of stress was determined by the number of all entries to the central squares and the time spent in the central part of the test. A bright, open space is a strong stressor for mice, so crossing the central fields of the box and staying there longer was considered as a sign of the animal’s courage (rodents with higher sensitivity to stress generally stay in the peripheral fields of the test). All categories of animal behavior were recorded using a camera and the Ethovision XT 10 software (Noldus, Wagenigen, the Nederlands) for 10 minutes, three times: before the start of administration, after one week and after fourteen days of administration of bacteriophage cocktail, antibiotic or saline.

### 2.6 Analysis of anxiety behavior and memory processes in the elevated plus-maze test

Similar to the above-described open field test, the elevated plus-maze test allows assessment of the anxiety response, based on the natural tendency of rodents to actively explore new environment, which is limited by the aversive properties of the elevated plus open part of the test. This test can also be used to record memory processes under anxiety conditions by recording the transfer latency from the open to the closed arm. In addition, the implementation of a re-test (repeat measurement) procedure, allows the evaluation of memory processes. The apparatus used to carry out the test, consisted of a cross-shaped (plus) platform raised about 50 cm above the ground. Two of the platform’s arms were shielded by walls, while two remained open. The dimensions of the arms were 5 x 10 cm, respectively. During the test, the procedure was carried out three times for each group. During the intervals, the apparatus was washed with 70% ethanol after each trial and allowed to dry for five minutes so that the smell of other animals would not affect the experiment. The first trial was considered as a baseline measurement, allowing to exclude the individuals whose results deviate from the average value for the group. In further stages of the experiment, the baseline measurement was also considered as a reference point. The second trial was performed after seven days of the administration onset, while the third trial was carried out after fourteen days of the treatment with the bacteriophage cocktail, antibiotics or saline. All trials were recorded using an analog camera and EthoVision XT 10 software (Noldus, Wageningen, the Netherlands). Reactions considered were: time spent in the open/closed arms; number of entries into the open/closed arms; as well as exploration and immobilization.

### 2.7 Blood collection

Blood was collected from mice at three time points: under baseline conditions (before the start of administration, but also before behavioral testing – baseline measurement) and after seven and then fourteen days from orally administration onset of the bacteriophage cocktail, antibiotics or saline. This procedure was performed under short-term ketamine (87.5 mg/kg body weight) and xylasine (12.5 mg/kg body weight) anesthesia from the *venous plexus* inside the orbit behind the eyeball. Blood was collected in a volume representing 6% of the animal’s body weight into EDTA-containing tubes using capillaries 2 cm in length, approximately 1 mm in diameter with the interiors coated with the same anticoagulant. Each blood sample collected was immediately divided according to the course of further determination: 700 µl of whole blood was used to obtain the results of flow cytometry and hematological parameters, while the remaining blood was centrifuged (10 minutes, 2000 × *g*, 4°C) to obtain plasma, which was subjected to deep freezing (-80°C) until further analysis.

### 2.8 Analysis of selected blood hematological parameters

The hematological analysis of previously collected whole blood (200 µl) was performed in a Horiba ABX Micros ES 60 automatic analyzer (Horiba Medical, Japan). Following parameters were monitored: number of leukocytes, lymphocytes, monocytes and granulocytes, as well as the red blood cell system indexes: erythrocyte count, hemoglobin (HGB) level, hematocrit (HCT) level, mean red cell volume (MCV), mean corpuscular hemoglobin (MCH), mean corpuscular hemoglobin concentration (MCHC), and platelet (PLT) number.

### 2.9 Determination of the percentage of lymphocyte population and subpopulations of T helper (Th, TCD4+) and T cytotoxic (Tc, TCD8+) in peripheral blood by flow cytometry

Cytometric analysis of the lymphocyte population was performed after centrifugation of blood in a Ficoll gradient (1,113 × *g*, 30 minutes, 4°C) and uropollin according to the procedure described previously (18). Peripheral blood mononuclear cells (PBMCs, mainly lymphocytes and monocytes), isolated by this method, were suspended at a final concentration of 10^7^ cells/ml. For cytometric verifications, 25 µl of prepared PBMC cell suspension and 25 µl of antibodies selected from two kits, AntiMouse CD3-FITC/CD45RA-PC7/CD161a-APC or CD3-FITC/CD4-PC7/CD8-APC (Beckman Coulter, California, USA), were employed. The samples were incubated for 20 minutes in the dark at room temperature. After incubation, 700 µl of buffered saline (PBS) and 25 µl of fixative solution (Fixative Solution IOTest O3, Beckman Coulter, California, USA) were added. The percentage of lymphocyte population and subpopulations was determined by flow cytometry using the FACSVerese cytometer (Becton Dickson) and BD FACSuite software version 1.0.5. The separation into subpopulations was based on the surface expression of CD4 (helper T cells, Th, TCD4+) or CD8 (cytotoxic T cells, Tc, TCD8+). The total number of lymphocytes and their subpopulations was calculated based on the total number of leukocytes and the percentage of T, TCD4+ and TCD8+ lymphocytes.

### 2.10 Determination of pro-inflammatory (IL-6, TNF-α) and anti-inflammatory (IL-10) cytokine concentrations in blood plasma

Plasma IL-6, TNF-α and IL-10 concentrations were determined by enzyme–linked immunoassay (ELISA) using a commercially available kit (My BioSource Inc., San Diego, USA) according to the manufacturer’s instructions and using a Multiskan Fc microplate reader (Thermo Fisher Scientific, Massachusetts, USA), coupled with Skanlt 6.1.1 RE software, which analyzes spectrophotometric color intensity, plots a standard curve based on the standards used, and reads the concentration values of the particular cytokines in the plasma samples tested. The results obtained are presented in pg/ml.

### 2.11 Mice weighing procedure

Mice were weighed three times: at the beginning of the experiment (before blood collection and behavioral tests performed under baseline conditions), then after seven and fourteen days after bacteriophage cocktail, antibiotic or saline administration onset, depending on the experimental group. After removal from the home cage, the animal was placed in a plastic container, 15 cm in diameter and 18 cm high, which was then placed on the scale (Soehlnc Professional, Nassau, Germany). The total duration of the activity did not exceed 30 seconds.

### 2.12 Mice euthanasia

Mice were given a lethal intraperitoneal dose of pentobarbital anesthesia at 120 mg/kg body weight, and internal organs were harvested. To minimize the animal’s discomfort during the procedure, immediately before the injection, the mouse was additionally anesthetized with isoflurane inhalation anesthesia (2.5%, flow rate 0.5 l/minute).

### 2.13 Weighing internal organs

Briefly, all organs: thymus, spleen, brain, kidney, heart, liver, intestines and stomach subjected to the weighing procedure were taken entirely from each animal. They were then purified (removal of residual fat) by washing in buffered saline solution (PBS). The intestines were cut off immediately after the stomach, at the level of the *pylorus*, and were taken as far as the *rectum*. Intestines before weighing were cleaned off any remaining digestive contents.

### 2.14 Preparation of homogenates

All organs were sliced with a sterile scalpel into smaller pieces, the size of which dependent on the tissue type. In the case of the brain and kidney, their fragments were 50 mg, the heart and spleen 5 mg, and the liver 100 mg. In order to remove any external contamination (blood, vessels, fat), the organs were rinsed three times in 1 ml PBS. Homogenization was performed using a Bullet Blender Tissue Homogenizer (Next Advance, NY, USA), according to protocols, dedicated to the specific tissue type. The appropriate type of grinding beads was added to the microcentrifuge tubes. In the case of brain and liver, glass beads (0.5 mm, product number GB05) were used. For the homogenization of the heart, stainless steel beads (1.6 mm, product number SSB16) were used. The zirconium oxide beads (0.5 mm, product number ZROB05) were used to homogenize the kidney and spleen. The weight of the beads used had to be equal to the weight of the homogenized organ. The next step was to add two volumes of homogenization buffer (T-PER Tissue Protein Extraction Reagent, Thermo Scientific, product number 78510, Massachusetts, USA), containing protease inhibitors (Thermo Scientific, product number A32955, Massachusetts, USA) for every 100 mg of organ. The organ prepared in this way was centrifuged for 5 minutes at maximum speed (level 12; 10,000 RPM), then the supernatant was collected and frozen until further analysis.

### 2.15 Isolation of total DNA

Homogenates from brains, hearts, livers, spleens and kidneys were used to isolate total DNA. RNase (final concentration 5 µg/µl; EURx, Poland) was added to 300 µl of the lysate and incubated at 37°C for 30 minutes. Next, thermal inactivation of RNase was performed for 10 minutes at 65°C. To samples obtained in this way, 400 µl of Tissue Cell Lysis Solution (Lucigen, USA) and 5 µl of Proteinase K (concentration 25 mg/ml; EURx, Poland) were added, and then incubated for 30 minutes at 65°C. Samples were cooled in ice for 5 minutes, then 300 µl MPC Protein Precipitation Reagent (Lucigen, USA) was added and centrifuged (8,000 x *g*, 10 minutes, 4°C). Five hundred µl of isopropanol (POCH, Poland) were added to the supernatant and incubated at -20°C for 24 hours. Then, the samples were centrifuged (9,600 x *g*, 20 minutes, 4°C), the supernatant was removed, and 700 µl of 70% ethanol (POCH, Poland) were added to the resulting colorless pellet. The samples were centrifuged (9,600 x *g*, 40 minutes, 4°C), the supernatant was removed, and 500 µl of 70% ethanol were added to the white pellet. The supernatant was removed, and the pellet was dried for 20 minutes under vacuum at 30°C. The pellet was suspended in 30 µl of nuclease free water (Roth, Germany) and incubated for 15 minutes at 37°C to dissolve. The obtained samples were stored at -20°C.

### 2.16 Primer design

Specific primers were designed by Primer-BLAST software, with parameters set to exclude *Mus musculus* (taxid: 10090), Caudoviricetes (taxid: 2731619), and Viral (taxid: 10239) sequences. Forward (5’AGCGTTAGTTCTGTCCACCC3’) and reverse (5’CGCTGGCACTAATTTCGGTG3’) primers of the length of 20 nucleotides are complementary to positions flanking the 37654-38634 nucleotides region of the *Salmonella* bacteriophage vB_Sen-TO17 genome, which encodes hypothetical tail and neck proteins. As for *Salmonella* bacteriophage vB_SenM-2, primers (Forward primer: 5’GCGCGACTTGTAAGATGCTG3’, Reverse primer: 5’CCAATCAAGGGGCTTCTCGT3’) were designed to target the nucleotide span 157621-157987 within the genome, encoding a hypothetical neck protein.

### 2.17 Bacteriophage DNA identification using PCR

The PCR reaction was performed for the identification of bacteriophage DNA. The reaction was performed using Color Taq PCR Master Mix (EURx, Poland), specific primers (listed in Section 2.15, Genomed, Poland), nuclease free water (Roth, Germany) and the matrix (isolated according to the section 2.14.). The reaction was conducted with the following parameters: denaturation – 15 seconds, 94°C; annealing – 15 seconds, 55°C; extension – 60 seconds, 72°C; number of cycles: 30.

### 2.18 Electrophoresis and gel visualization

The obtained PCR reaction products were visualized in a 1.5% agarose gel (agarose solution in Tris-Octane-EDTA buffer (Bioshop, Canada) supplemented with SimplySafe™ (EURx, Poland) solution according to the manufacturer’s instructions). Electrophoresis was conducted for 30 minutes at 100 V. The gels were then visualized using a gel documentation system (FastGene FAS-DIGI PRO, Nippon Genetics Europe, Germany). Parameters of the images taken: aperture 9 AV, exposure 1/50 TV, ISO 1600.

### 2.19 Determination of bacteriophage numbers in mouse organs

Ten μl of the lysate of the appropriate organ was diluted in 90 μl of 0.89% NaCl. Serial dilutions (1:9 v/v each) were then prepared in 0.89% NaCl. Then, 100 μl of each dilution were added to 200 μl of the overnight culture of *Salmonella* Typhimurium in LB medium and incubated for 10 min, to adsorb bacteriophages at the bacterial surface. Then, 4 ml of the Taq medium (0.7% bacteriological agar (BTL, Poland) in LB medium (Bioshop, Canada) was added and poured into Petri dishes with LB-agar medium (1% Agar-Agar (BTL, Poland) in LB medium). The plates were incubated at 37°C for 24 hours. The bacteriophage titer was then counted on the basis of number of plaques appearing on plates. The bacteriophage titer was then counted according to the formula: PFU/g = A × $\frac{1000}{V}$ × 10^n^, where: A - plaque number in a particular plate, V - volume of bacteriophage stock, 10^n^ - bacteriophage dilution factor.

### 2.20 Microbiome analysis

Bacterial genomic DNA extracted from the gastrointestinal tract was purified according to the method described previously (12), using a commercially available reagent kit (Invitrogen, Carlsbad, CA, USA). The obtained DNA samples were sent (Genomed S.A., Warsaw, Poland) for 16S rRNA gene PCR amplification, library preparation, illumina MiSeq sequencing, and bioinformatics taxonomy analysis. The Shannon diversity index, taking into account the OTU (the abundance of each operational taxonomic unit) value, was calculated using the PAST software version 4.09. The Shapiro-Wilk test was used to determine the normality of the diversity index data, and a comparison of the variability of the bacterial families in the experimental groups and the control group was carried out using the paired samples T-test. The final step was to perform a frequency analysis and the chi square test to determine the relative contribution of the particular bacterial families in the microbiome of each experimental group. All the aforementioned comparisons were performed using IBM SPSS 21.0 software (SPSS Inc., Amonk, USA).

### 2.21 Statistical analysis

The results are presented as mean ± standard deviation (SD). For statistical analyses of the results, SPSS 21.0 (SPSS Inc., Amonk, USA) software was used. The normality of the distribution of variables was checked with the Kolmogorov-Smirnov test, and the homogeneity of the variances with the Levene test. When the outcome of the Kolmogorov-Smirnov test indicated that the data were not distributed normally, we used non-parametric Kruskal-Wallis and Dunn tests for further analysis. For other parameters, two-way ANOVA and Tukey’s *post hoc* tests were performed. The *p* value lower than 0.05 was considered statistically significant.

## 3 Results

### 3.1 Antibiotic therapy induces hyperactivity in the actometers which is more pronounced in females

**Figure 2:**
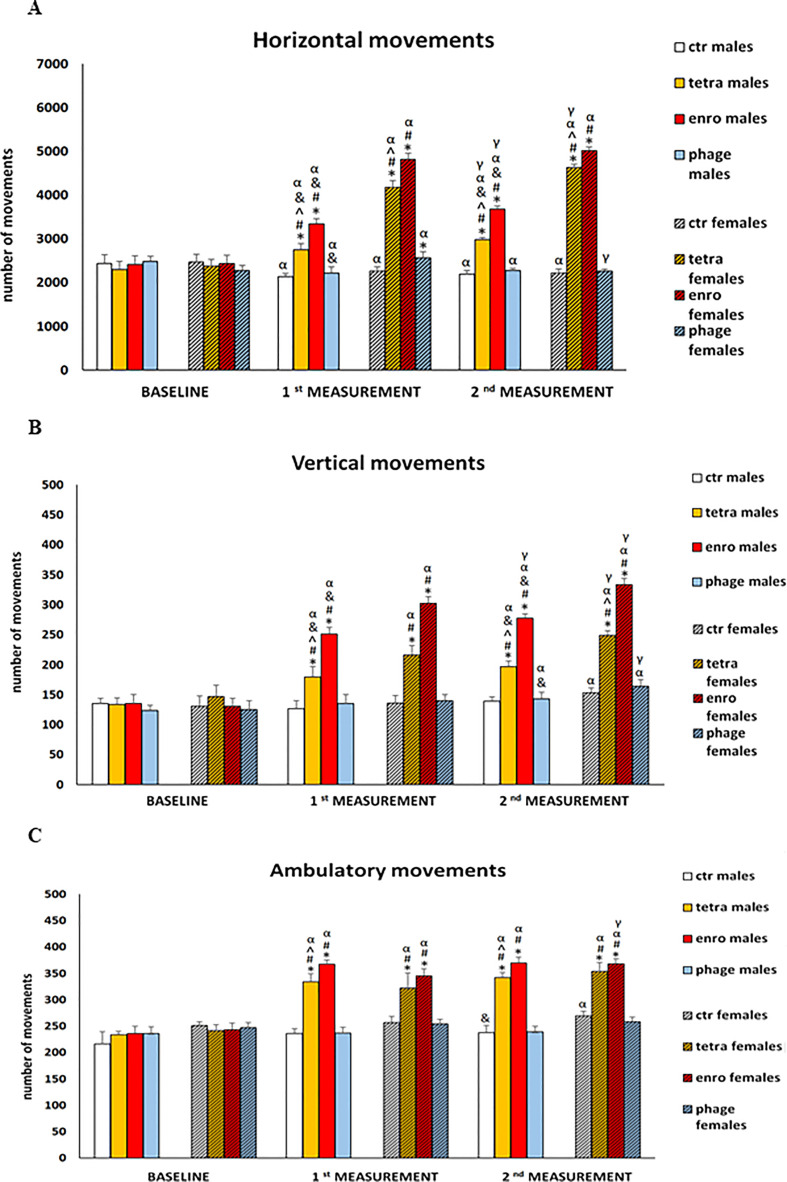
Changes in the number of three types of movements performed in 10 minutes in actometers: **(A)** horizontal, **(B)** vertical and **(C)** ambulatory in male and female mice receiving saline, antibiotics or bacteriophage cocktail. Results are presented as mean values ± SD. Statistical analyses were performed by ANOVA and *post-hoc* Tukey test for horizontal and vertical movements and by Kruskal-Wallis test and *post-hoc* Dunn test for ambulatory movements. The significance of differences between controls and particular treated groups are marked by: asterisks (*) vs. saline control males or saline control females group; (#) vs. bacteriophage males or bacteriophage females group; (^) vs. enrofloxacin males or enrofloxacin females group; (&) vs. females; (α) vs. baseline value; (γ) vs. 7 days.

To test effects of administrations of the bacteriophage cocktail and antibiotics on functions of the central nervous system (memory and learning processes, anxiety reactions, locomotor activity), behavioral assays were performed. Number of horizontal, vertical and ambulatory movements were determined at various times during the treatment (**Figure 2**). Unexpectedly, we found that antibiotic therapy led to severe behavioral disturbances, already after seven days of the administration, which were manifested by hyperactivity, expressed by an increased number of the three types of movement. The observed deviation from the natural behavioral pattern persisted throughout the whole antibiotic treatment period (fourteen days). Interestingly, this feature was significantly more severe in females than in males. Such a disturbed behavioral phenotype may be an indication of a seriously impaired central nervous system function, following the use of antibiotics, particularly the enrofloxacin. In contrast, both males and females receiving the bacteriophage cocktail did not differ in their locomotor behavior from animals in the control groups throughout the supplementation period.

### 3.2 Antibiotics generate anxiety behaviors that are more severe in females

**Figure 3:**
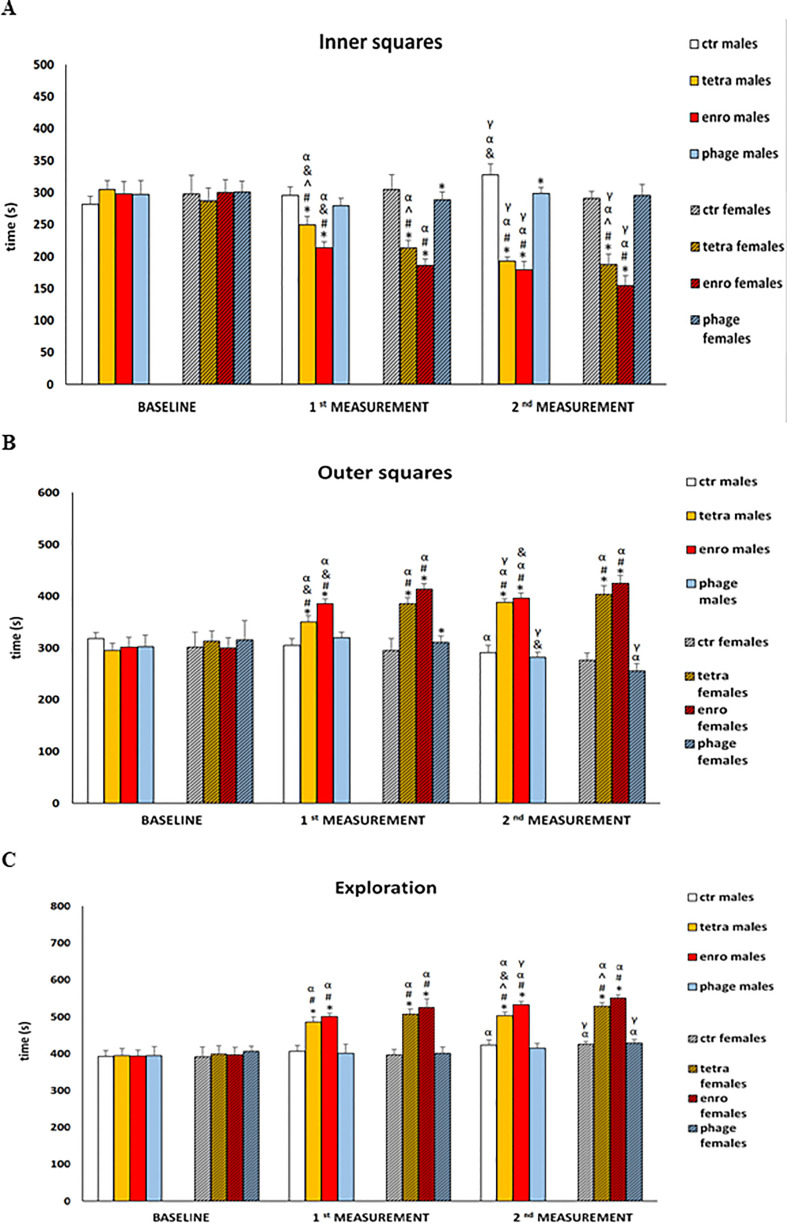
Changes in the anxiety behavior in the open field test (10-minute recording): **(A)** time spent in the inner squares, **(B)** time spent in the outer squares, **(C)** exploration in male and female mice receiving saline, antibiotics or bacteriophage cocktail. Results are presented as mean values ± SD. Statistical analyses were performed by ANOVA and *post-hoc* Tukey test. The significance of differences between controls and particular treated groups are observed and marked by: asterisks (*) vs. saline control males or saline control females group; (#) vs. bacteriophage males or bacteriophage females group; (^) vs. enrofloxacin males or enrofloxacin females group; (&) vs. females; (α) vs. baseline value; (γ) vs. 1^st^ measurement value.

The results of anxiety behavior in the open field test are shown in **Figure 3**, and in **Supplementary Figures S1**, **S2**. Another indication of antibiotic-mediated central nervous system dysfunction was an increase in the anxiety behavior. The central squares, which are open spaces that are further illuminated by bright light, are a factor for rodents to induce severe stress and anxiety. Animals that, despite the aversive nature of this test zone, stay in it for a longer period of time are characterized by lower levels of anxiety. High level of stress was mainly manifested by shorter duration of stay, fewer entrances, and shorter distance travelled in the central (inner) quadrants by mice. In addition, the administration of antibiotics, especially enrofloxacin, caused hyperactivity similar to that observed in actometers. All of these disturbances were evident after only seven days of administration and, noteworthy, they were particularly severe in females. In contrast, the behavior of both males and females receiving the bacteriophage cocktail did not differ from that of the control groups and did not show symptoms of increased anxiety.

### 3.3 Short-term memory impairment following antibiotics administration

**Figure 4:**
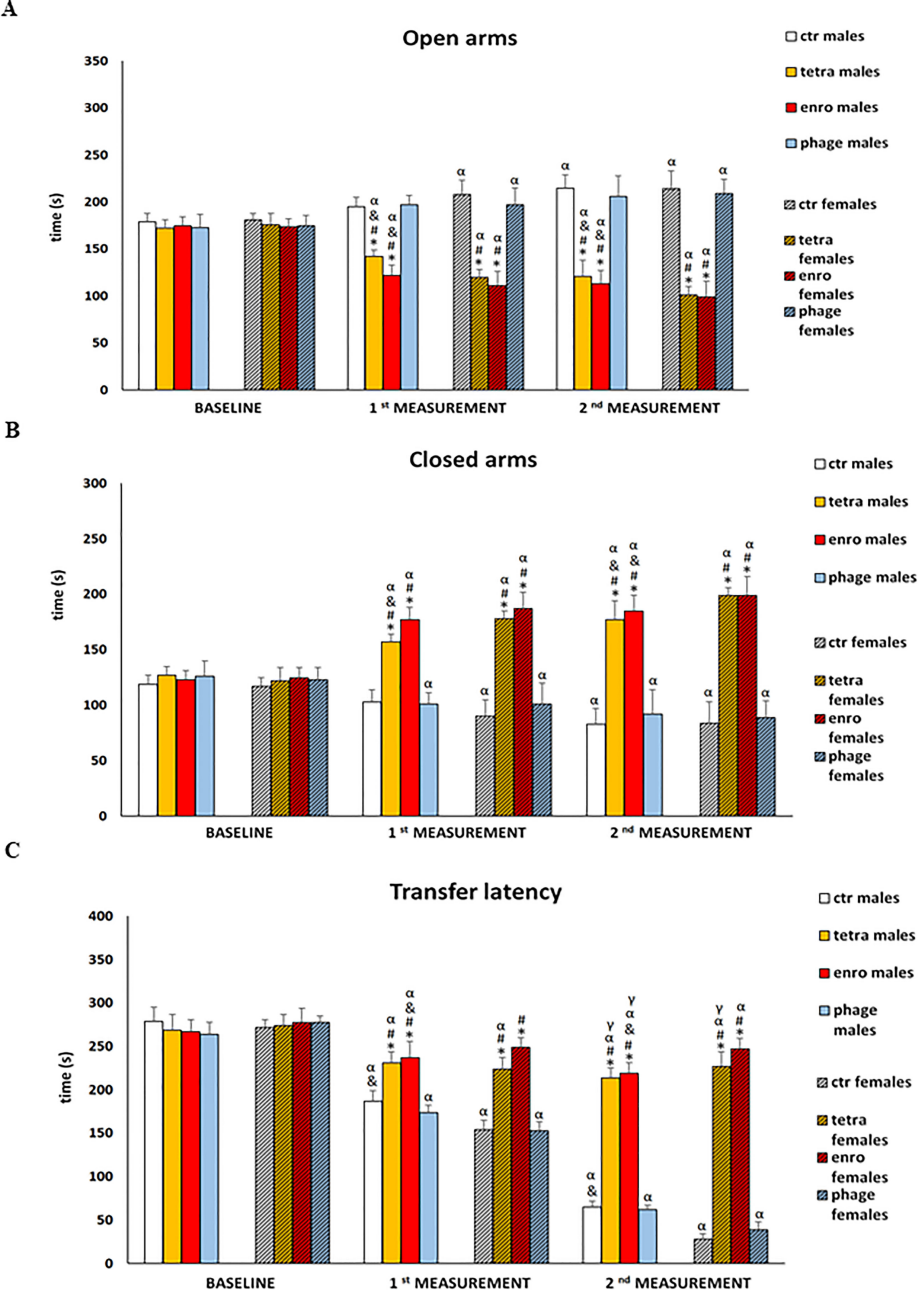
Changes in the anxiety behavior and memory processes in the elevated plus-maze test (5 minutes): **(A)** time spent in the open arms, **(B)** time spent in the closed arms, **(C)** transfer latency in male and female mice receiving saline, antibiotics or bacteriophage cocktail. Results are presented as mean values ± SD. Statistical analyses were performed by Kruskal-Wallis test and *post-hoc* Dunn test. The significance of differences between controls and particular treated groups are observed and marked by: asterisks (*) vs. saline control males or saline control females group; (#) vs. bacteriophage males or bacteriophage females group; (&) vs. females; (α) vs. baseline value; (γ) vs. 1^st^ measurement value.

The results of assessment of the anxiety level and memory processes in the elevated plus-maze test (EPM) are presented in **Figure 4** and **Supplementary Figure S3**. Analysis of the rate of movement of mice from the open, aversive arm to the closed (safe) arm makes it possible to study the course of working (short-term) memory, which is disrupted not only in the course of neurodegenerative diseases, but also under the influence of strong stimuli, such as stress/anxiety. A more complex behavioral analysis, including not only anxiety levels but also memory processes, carried out in the EPM test, confirmed previous observations of nervous system dysfunction after antibiotic therapy. Statistical analysis after just seven days of the administration onset showed significantly reduced number of entries and shorter time spent in the open arms of the maze by mice. Furthermore, both antibiotics (though especially enrofloxacin) interfered with appropriate learning and memory processes, expressed by a prolongation of the transfer latency from the aversive (open) to the closed (safe) arms. In addition, non-natural locomotor activity was noted. All these abnormalities persisted throughout the supplementation period and were significantly more severe in females. For the group receiving the bacteriophage cocktail, both male and female mice did not differ from the control animals in the analyzed parameters.

### 3.4 Antibiotic therapy results in leukocytosis which is more severe in males

The absolute numbers and relative numbers of leukocytes, lymphocytes, monocytes, as well as red blood cell parameters, measured in all tested groups, are shown in **Supplementary Table S1** and in the **Supplementary Table S2**. The absolute number of leukocytes (especially lymphocytes, but also granulocytes and monocytes) was elevated after tetracycline and enrofloxacin supplementation. Elevated lymphocyte levels indicate chronic or severe bacterial/viral infections, the development of inflammation, dehydration or neurological injury. In turn, increased granulocyte production occurs during inflammation. Elevated monocytes often appear after past infections, at a time when there is an intense renewal of leukocytes after infection. In contrast to behavioral studies, the negative effects of the antibiotic therapy on hematological parameters were more severe in males than in females. When the bacteriophage cocktail was used, the values observed did not differ significantly from those noted in the control groups. The same conclusion applied to the relative values. As for erythrocyte indices, the statistically significant reduction was noted in both females and males after administration of both antibiotics. Such results may indicate the initial phase of anaemia, but also bone marrow failure.

### 3.5 Decrease in the percentage of T lymphocytes and their key subpopulations (Tc, TCD8+ and Th, TCD4+) after antibiotic therapy

**Figure 5:**
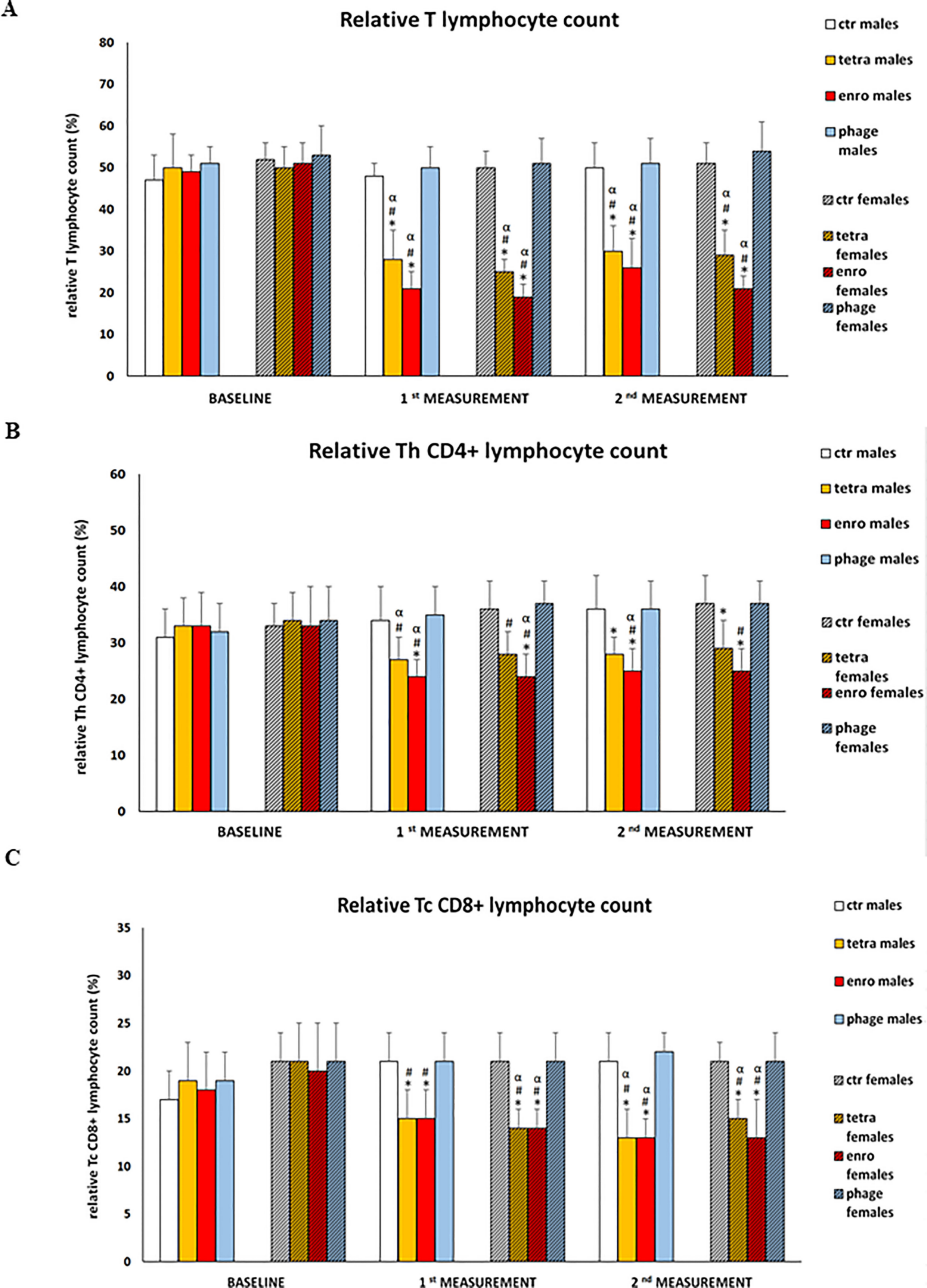
Changes in relative T lymphocyte **(A)**, relative Th TCD4+ **(B)** and relative Tc TCD8+ **(C)** counts in the blood of male and female mice receiving saline, antibiotics or bacteriophage cocktail. Results are presented as mean values ± SD. Statistical analyses were performed by ANOVA and *post-hoc* Tukey test. The significance of differences between controls and particular treated groups are observed and marked by: asterisks (*) vs. saline control males or saline control females group; (#) vs. bacteriophage males or bacteriophage females group; (&) vs. females; (α) vs. baseline value.

The results of cytometric analyses are presented in **Figure 5**. Interestingly, these analyses showed a statistically significant reduction in the percentage of T lymphocytes, as well as key cytotoxic and T helper subpopulations, in antibiotic-treated animals. This adverse effect of the antibiotic therapy (which was more severe after enrofloxacin administration) was seen at comparable levels in animals of both sexes. An insufficient proportion of T cytotoxic lymphocytes may hinder the elimination of cells infected by viruses or other intracellular parasites. In turn, a deficiency of T helper lymphocytes may reduce the release of cytokines, which are important mediators of differentiation and antibody release by B lymphocytes. We noted the opposite effect after administration of bacteriophages, namely, such a treatment did not affect the percentage of key immune cells in mice.

### 3.6 Antibiotic therapy results in cytokine imbalance which is more severe in females

**Figure 6:**
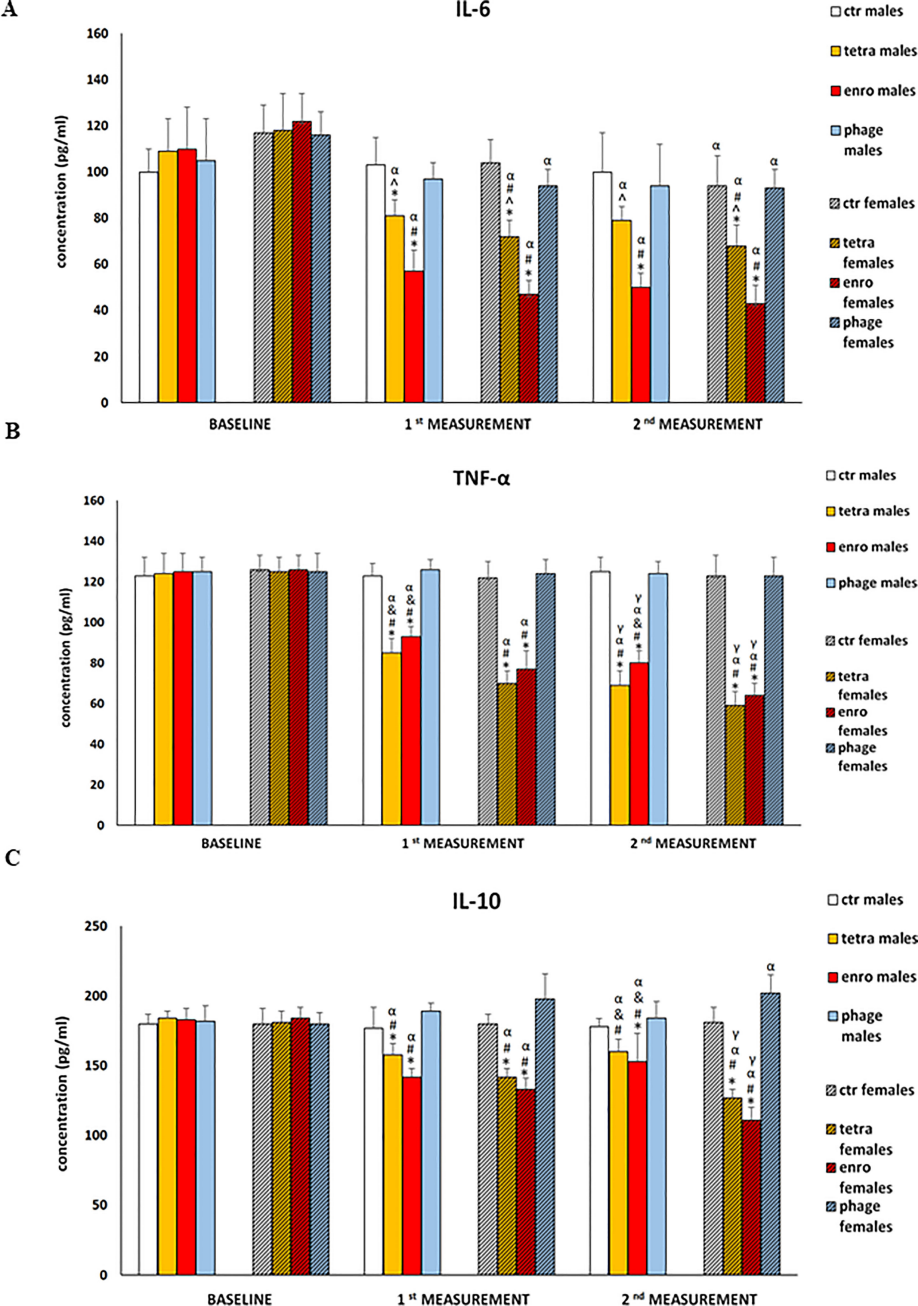
Changes in the cytokine concentrations: Il-6 **(A)**, TNF-α **(B)**, and IL-10 **(C)** in the blood plasma of male and female mice receiving saline, antibiotics or bacteriophage cocktail. Results are presented as mean values ± SD. Statistical analyses were performed by ANOVA and *post-hoc* Tukey test. The significance of differences between controls and particular treated groups are observed and marked by: asterisks (*) vs. saline control males or saline control females group; (#) vs. bacteriophage males or bacteriophage females group; (&) vs. females; (α) vs. baseline value; (γ) vs. 1^st^ measurement value.

The results of measurements of pro-inflammatory (IL-6, TNF-α) and anti-inflammatory (IL-10) cytokine concentrations in plasma are presented in **Figure 6**. Statistical analysis showed a significant cytokine imbalance induced by the administration of both antibiotics. As with most other parameters, this negative effect was especially pronounced during enrofloxacin supplementation, and showed a greater severity in females. The changes consisted of not only a decrease in the concentration of both pro-inflammatory cytokines (TNF-α and IL-6), but also IL-10. One should note that TNF-α and IL-6 are particularly important for the regulation of memory processes, and IL-10 exerts an anti-inflammatory effect. In contrast, the use of bacteriophages did not induce any changes in levels of investigated cytokines.

### 3.7 Weight loss following the antibiotics administration

The average weight of the mice at the start of the experiments was 29 ± 2 g and 25 ± 2 g for males and females, respectively. After fourteen days of saline or bacteriophage cocktail supplementation, there was an increase in the weight of mice by an average of 2 g, while the antibiotic treatment groups showed a statistically significant decrease in weight by an average of 2 (tetracycline) or 4 (enrofloxacin) g.

### 3.8 Antibiotic therapy leads to a reduction in organ weights which is more severe in females

The results of the weight of particular internal organs after fourteen days of antibiotic or bacteriophage administration are presented in the **Supplementary Figure S4**. Statistically significant reduction in weight was seen in the spleen, thymus, kidneys, intestines and stomach. In all of these organs, the reduction of weight was evident after the antibiotic therapy, particularly when the administration of enrofloxacin was performed, and there was more pronounced in females. Interestingly, the opposite situation was found for the heart and liver, as after the antibiotic therapy, there was an increase in heart and liver weight in females and in mice of both sexes treated with enrofloxacin. Only for the brain, it was no difference in the organ weight between groups and sexes. In the groups receiving the bacteriophage cocktail, the organs’ weights did not differ from those in the control groups.

### 3.9 Confirmation of the presence of bacteriophages in the examined organs

Identification of bacteriophages in organs of mice treated with the bacteriophage cocktail was performed by PCR. The organs were homogenized, and then total DNA was isolated.

**Figure 7:**
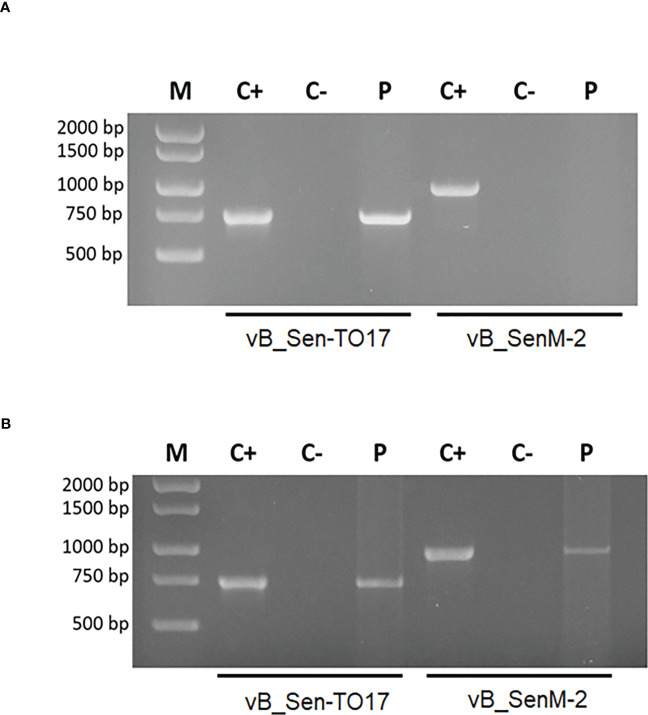
Identification of bacteriophages in brains of male **(A)** and female **(B)** mice treated with the bacteriophage cocktail. Specific products of 988 bp (bacteriophage vB_SenM-2) and 736 bp (bacteriophage vB_Sen-TO17) were analyzed by the PCR method. The matrix in the positive control was DNA isolated from purified bacteriophage lysate (PFU/ml=10^9^). For the negative control, water was added instead of matrix.

Specific products of 988 bp (bacteriophage vB_SenM-2) and 736 bp (bacteriophage vB_Sen-TO17) were confirmed in the brains of female mice, however, no specific product was obtained for bacteriophage vB_SenM-2 in the brains of male mice (**Figure 7**). Moreover, products specific for both bacteriophages were obtained in spleens, livers and kidneys of males and females. However, products specific for bacteriophages vB_SenM-2 and vB_Sen-TO17 were observed in male hearts, while they were not detected in hearts of female mice (**Supplementary Figure S5**).

The number of bacteriophages in the liver, spleen, kidney, heart and brain was also determined by the titration method. The presence of bacteriophage vB_Sen-TO17 in the liver, spleen, kidney, heart and brain of females and males was found. Interestingly, the bacteriophage vB_SenM-2 was present in the livers, spleens, kidneys and hearts of males. The titer of bacteriophage vB_SenM-2 was 5 times lower than that of bacteriophage vB_Sen-TO17 in the livers of females and 2.5 times lower in the livers of males. In the kidneys and spleens, the titer of bacteriophage vB_Sen-TO17 was 10 times higher than that of bacteriophage vB_SenM-2 in females and males. In addition, the presence of bacteriophages vB_Sen-TO17 and vB_SenM-2 in the brains of females was noted. However, bacteriophage vB_SenM-2 was not detected in the brains of males.

### 3.10 Microbiome changes following bacteriophage and antibiotic administration

**Figure 8:**
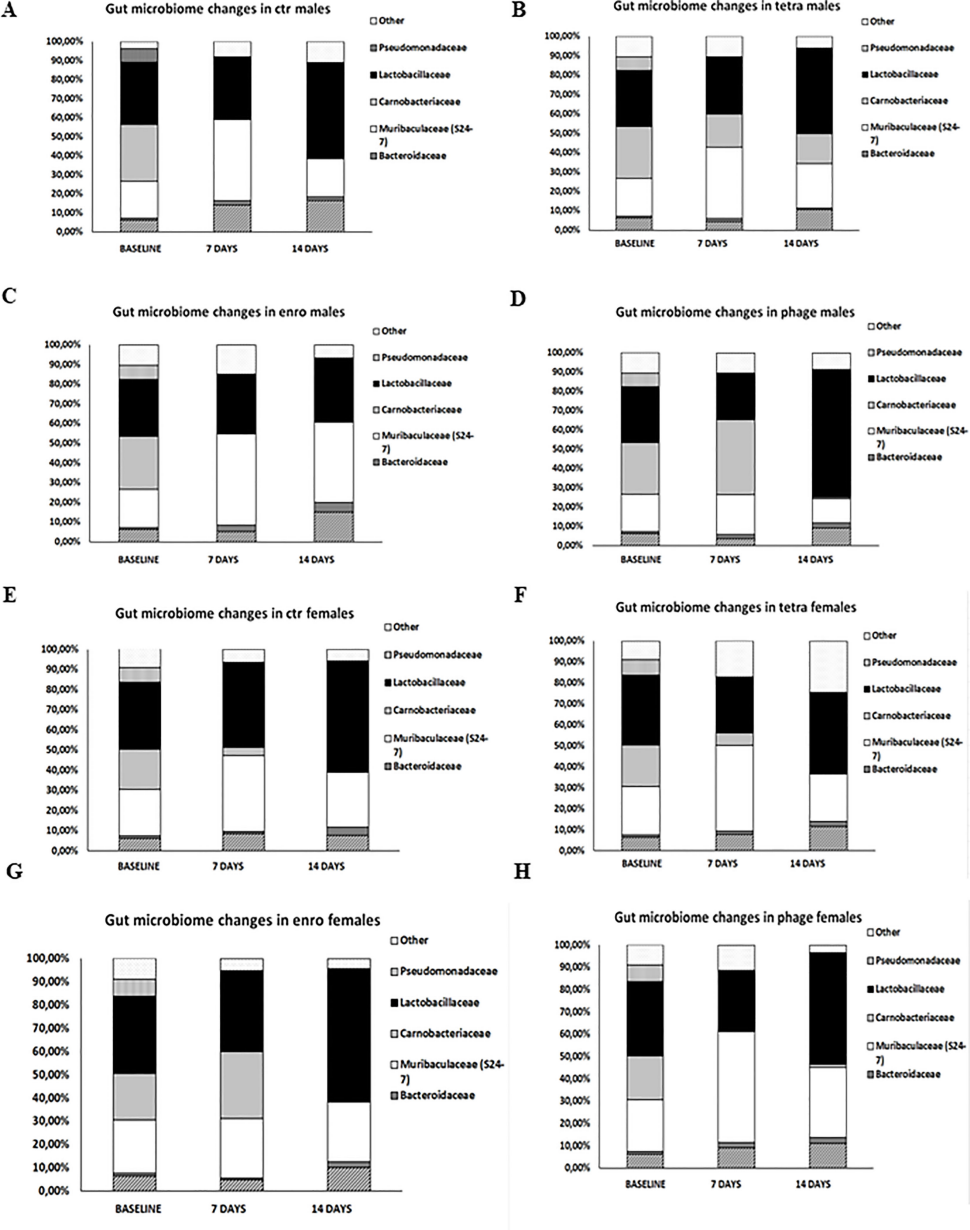
Differences in mice intestinal microbiome in particular groups: **(A)** males saline control group; **(B)** males tetracycline group; **(C)** males enrofloxacin group; **(D)** males bacteriophage group; **(E)** females saline control group; **(F)** females tetracycline group; **(G)** females enrofloxacin group; **(H)** females bacteriophage group.

As indicated in **Figure 8** and **Supplementary Figure S6**, the most significant changes in the microbiome were observed after fourteen days of the antibiotic or bacteriophage supplementation, respectively. Interestingly, after treatment with enrofloxacin, *Muribaculaceae* predominated in males. In contrast, in bacteriophage-treated females, this bacterial family dominated after only seven days of supplementation. In turn, enrofloxacin-treated females had a microbiome dominated by bacteria from the *Lactobacilaceae* family. Similar distribution of bacteria was observed in mice of both sexes from the control groups and those receiving bacteriophage cocktail.

## 4 Discussion

In the present study, we compared effects of administration of antibiotics (enrofloxacin and tetracycline) and bacteriophage therapy in a mouse model. We demonstrated, for the first time, a sex-dependent negative effect of antibiotic therapy, which not only involved the functioning of the immune system, but also significantly impaired the activity of the central nervous system, as manifested by a disruption of the behavioral pattern, particularly exacerbated in females. On the other hand, the complex behavioral and immunological analyses confirmed the lack of adverse effects after the bacteriophage cocktail administration.

The issue concerning the differences in the presence of bacteriophages in the various organs in males and females undoubtedly requires further research. Nevertheless, it can be speculated that, as with the bioavailability of nanomedicines, the differences between the sexes are on the cellular and molecular levels. Physiological differences between the sexes are also not limited to body fat and water content, plasma volume or the amount of blood reaching particular organs. For example, it has been confirmed that there are differences between men and women in the expression of thousands of genes that determine the functions of the liver, adipose tissue or skeletal muscle. In turn, the kidneys showed the presence of transporters which expression levels differ between males and females. Transcriptomic analysis of human kidneys confirmed the presence of twenty-one genes with male dominance and two transporter genes with female dominance. Further differentiating factors which role should not be overlooked are sex hormones (19). Moreover, analyses carried out on the heart showed that the profiles of functionally relevant proteins and their isoforms differed in animals of both sexes. These differences, included more than twenty-two proteins and increased significantly with the age of the mice (20). Male-specific expression of Y-linked genes was observed not only in mouse heart, but also in the human myocardium (e.g. Ddx3y, Eif2s3y and Jarid1d). Higher expression levels of X-linked genes were detected in female mice for Xist, Timp1, Car5b, XIST, EIF2S3X and GPM6B. In addition, genes on autosomal chromosomes encoding cytochromes of the monoxygenase family (e.g. Cyp2b10), carbonic anhydrases (e.g. Car2 and Car3) and natriuretic peptides (e.g. Nppb) were identified with sex- and/or age-specific expression levels (21). Furthermore, only female mice showed differences in the expression of important genes, including those regulating DNA metabolism, which showed a strong dependence on tissue/organ type following exposure to low doses of radiation (22). What is more, previous studies have shown that DNA isolates obtained from different types of organs from female mice confirmed significant differences in the level of damage as a result to exposure to organic wood preserving waste extracts. Adduct profiles were tissue-specific and displayed a multitude of non-polar DNA (23). Interestingly, the response to viral infection is strongly dependent on hormonal regulation and differs between males and females. Experiments with non-gonadectomized rats have shown that infection with Seoul virus results in the elevated levels of viral RNA, which was detected in males, but not in females. In contrast, removal of the gonads in males resulted in comparable levels of viral RNA to that observed in intact females. The opposite effect was noticed in females, in which the levels were significantly higher. Induction of pattern recognition receptors (PRRs, TLR7 and Rig-1), expression of antiviral genes (Myd88, Visa, Jun, IRF7, IFNβ, Ifnar1, Jak2, Stat3 and Mx2), and production of Mx protein were elevated in the lungs of intact females compared with intact males. Hormone cycle activity appears to have a significant impact primarily on the induction of PRRs than downstream IFNβ or Mx2 expression (24). However, the mechanism underlying the differences in bacteriophage location in particular organs in animals of both sexes requires additional investigation. Although sex-dependent, central nervous system-related differences in response of animals to treatment with antibiotics were not – to our knowledge - described previously, indications of changed behaviors after antibiotic therapy were reported recently. Namely, it was showed that administration of clindamycin and/or amoxicillin caused severe behavioral disturbances (25). First of all, a deterioration of cognitive processes in the novel object recognition test and an increase in the percentage of depressive behavior episodes were noted in the tail suspension test. In contrast to our study, those experiments were conducted only with females. It was proposed that an indirect cause of the observed behavioral disturbances was a dysbiosis of the gut microbiome induced by antibiotics (11). One should note that such adverse effects may increase the risk of neurodegenerative diseases in the long term (26). Similar conclusions were included in the report describing investigations of the effects of antibiotic therapy on anxiety behavior in mice (27). Although the authors of that report also did not take into account differences between animals of both sexes, they observed that streptomycin treatment significantly increased anxiety in mice in the light-dark box test and in the elevated plus-maze test. Again, they also suggested a dysbiosis of the gut microbiome as a potential cause of the observed disturbances (27). The long-term effects of the low-dose penicillin intake by pregnant mice on these animals and their offspring shortly after the birth were also studied (28). Gut microbiome, blood-brain barrier permeability, central (brain) cytokine expression and behavior were analyzed to demonstrate that the use of antibiotics at an early stage of development can have negative and long-term side effects (28). Among other changes, a disruption of cytokine expression in the frontal cortex, which directly translated into behavioral disturbances (manifested by increased aggression, anxiety, and decreased social interaction), was evident. The behavioral pattern of the antibiotic-treated animals resembled autism spectrum disorders in children (28). Therefore, one might speculate that the lack of an adequate diversity of beneficial bacteria forming a part of the gut microbiome increases the permeability of the blood-brain barrier, thus negatively affecting the microglia immune response, myelination, the neurochemical structure of the brain or the activity of the hypothalamic-pituitary-adrenal stress axis (28).

In the case of our study, the most pronounced change in the microbiome was the increase in the percentage of bacteria from the still poorly understood *Muribaculaceae* family, both in males after enrofloxacin treatment and in females receiving the bacteriophage cocktail. Previous studies have shown that the *Muribaculaceae* family is associated with the formation of the inner mucus layer in the colon and the proper functioning of the intestinal barrier, and its abundance was strongly correlated with the level of propionate, a kind of short-chain fatty acid negatively correlating with the colorectal cancer in mice (29). In addition, these bacteria are important in adaptation to hypoxia-induced stress and in response to the inflammatory process (30). Although we did not analyze the central immune response, the cytokine imbalance we demonstrated in the plasma in mice after the antibiotic therapy might be an indirect indicator of the negative effects of the tested antibiotics on neuroimmune parameters. We demonstrated that as short as two-week treatment with antibiotic may result in a severely disturbed behavioral pattern. Therefore, it is tempting to speculate that repeated or prolonged administration of antibiotics or their use early in life might have negative consequences in the form of metabolic disorders, allergies or neurodegenerative diseases.

Obviously, there are limitations of our studies. The foremost one is that the central immune response and histological changes in the brain were not analyzed, and the persistence and severity of the observed abnormalities were not verified. Only in the case of the female heart both methods used did not give a conclusive result. As the plaque counting method allows the detection of viable bacteriophages and the results indicated a low number of them in the heart of females, the PCR performed from the deep-frozen material proved to be an insensitive method with too many limitations in this experimental scheme. The detection limit of PCR method is usually in the range of 10^3^- 10^5^ PFU/ml. Furthermore, for the procedure we carried out, no additional steps were used to increase the sensitivity, such as magnetic capture hybridization, which could have contributed to discrepant results with the titration method. Nevertheless, the presented results are important indications of the risks that the antibiotic use may entail. In addition, to our knowledge, this is the first demonstration that the gender factor can be included in such a complex analysis as an important determinant, conditioning the course of the immune and behavioral response to the administered compounds. The mechanism of the differences between males and females in appearance of adverse effects, related to the behavioral and immune functions, in the response to antibiotic treatment remains to be elucidated. One might imagine that differences in hormones and/or different permeability of the blood-brain barrier can be important factors, however, extensive studies are required to find the real reason(s). Nevertheless, it is also important to note that this study confirmed the general safety of the use of bacteriophages *in vivo* which is a promising sing in the light of potential approval of bacteriophage therapy as a therapeutic procedure that might be used in clinical practice and in veterinary use.

## Data availability statement

The datasets presented in this study can be found in online repositories. The names of the repository/repositories and accession number(s) can be found below: NCBI database, BioProject ID PRJNA967510.

## Ethics statement

The animal study was reviewed and approved by the Local Ethics Committee for Experimental Animals in Bydgoszcz (permission number 02/2022, dated on January 19, 2022).

## Author contributions

ŁG prepared the bacteriophage cocktail, participated in behavioral tests and sections of mice, prepared blood for future experiments, performed the analysis of levels of cytokines in mice blood plasma, performed the analysis of levels of blood morphological parameters, analyzed the percentage of lymphocytes in peripheral blood, performed the PCR reactions, statistical analysis, co-drafted the manuscript, and prepared the visualization of the results. GW participated in sections of mice and participated in analyses of results and co-drafted the manuscript. AW participated in sections of mice, participated in the analysis of levels of cytokines, analyzed data, and co-drafted the manuscript. KP participated in behavioral tests, analyzed data and co-drafted the manuscript. KK-K and MS participated in preparing the material for the microbiome analysis. MG participated in designing the primers. GJ participated in behavioral tests. MP presented the concept of the study, planned and coordinate experiments, participated and coordinated the sections of mice, participated in the behavioral analysis and analysis of levels of cytokines in mice blood plasma, participated in the analysis of levels of blood morphological parameters and participated in the analysis of the percentage of lymphocytes in peripheral blood, and co-drafted the manuscript. All authors contributed to the article and approved the submitted version.

## Supplementary material

Supplementary Figure 1 Changes in the anxiety behavior in the open field test (10 minutes): **(A)** frequency of entries to the inner squares; **(B)** frequency of entries to the outer squares; **(C)** immobility in male and female mice receiving saline, antibiotics or bacteriophage cocktail. Results are presented as mean values ± SD. Statistical analyses were performed by ANOVA and *post-hoc* Tukey test. The significance of differences between controls and particular treated groups are observed and marked by: asterisks (*) vs. saline control males or saline control females group; (#) vs. bacteriophage males or bacteriophage females group; (^) vs. enrofloxacin males or enrofloxacin females group; (&) vs. females; (α) vs. baseline value; (γ) vs. 1^st^ measurement value.

Supplementary Figure 2 Changes in the anxiety behavior in the open field test (10 minutes): **(A)** distance in the inner squares; **(B)** distance in the outer squares in male and female mice receiving saline, antibiotics or bacteriophage cocktail. Results are presented as mean values ± SD. Statistical analyses were performed by ANOVA and *post-hoc* Tukey test. The significance of differences between controls and particular treated groups are observed and marked by: asterisks (*) vs. saline control males or saline control females group; (#) vs. bacteriophage males or bacteriophage females group; (^) vs. enrofloxacin males or enrofloxacin females group; (&) vs. females; (α) vs. baseline value.

Supplementary Figure 3 Changes in the anxiety behavior and memory processes in the elevated plus-maze test (5 minutes): **(A)** number of entries to open arms, **(B)** number of entries to closed arms, **(C)** exploration in male and female mice receiving saline, antibiotics or bacteriophage cocktail. Results are presented as mean values ± SD. Statistical analyses were performed by ANOVA and *post-hoc* Tukey test for number of entries to open arms and exploration and by Kruskal-Wallis test and *post-hoc* Dunn test for number of entries to closed arms. The significance of differences between controls and particular treated groups are observed and marked by: asterisks (*) vs. saline control males or saline control females group; (#) vs. bacteriophage males or bacteriophage females group; (&) vs. females; (α) vs. baseline value; (γ) vs. 1^st^ measurement value.

Supplementary Figure 4 Changes in the weights of particular internal organs: **(A)** brain, **(B)** spleen, **(C)** thymus, **(D)** heart, **(E)** liver, **(F)** kidneys, **(G)** intestines and **(H)** stomach, after fourteen days of antibiotic or bacteriophage administration in male and female mice. Results are presented as mean values ± SD. Statistical analyses were performed by ANOVA and *post-hoc* Tukey test. The significance of differences between controls and particular treated groups are observed and marked by: asterisks (*) vs. saline control males or saline control females group; (#) vs. bacteriophage males or bacteriophage females group; (&) vs. females.

Supplementary Figure 5 Identification of bacteriophages in particular organs: **(A)** livers of male, **(B)** livers of female, **(C)** kidneys of male, **(D)** kidneys of female, **(E)** spleens of male, **(F)** spleens of female, **(G)** hearts of male, **(H)** hearts of female mice treated with the bacteriophage cocktail. Specific products of 988 bp (bacteriophage vB_SenM-2) and 736 bp (bacteriophage vB_Sen-TO17) were analyzed by the PCR method.

Supplementary Figure 6 The Shannon diversity index, taking into account the OTU (the abundance of each operational taxonomic unit) value.

Supplementary Table 1 Changes in the absolute (10^3^/µl) counts of leukocytes, lymphocytes, monocytes and granulocytes and relative (%) values of lymphocytes, monocytes and granulocytes in blood of male and female mice receiving saline, antibiotics or bacteriophage cocktail. Results are presented as mean values ± SD. Statistical analyses were performed by Kruskal-Wallis test and *post-hoc* Dunn test. The significance of differences between controls and particular treated groups are observed and marked by: asterisks (*) vs. saline control males or saline control females group; (#) vs. bacteriophage males or bacteriophage females group; (&) vs. females; (α) vs. baseline value.

Supplementary Table 2 Changes in the erythrocyte number (10^6^/mm^3^), hemoglobin (g/dL) and hematocrit (%) level as well as erythrocyte indicators: MCV (Mean Corpuscular Volume, fL), MCH (Mean Corpuscular Hemoglobin, pg), MCHC (Mean Corpuscular Hemoglobin Concentration, g/L) and platelet number (10^3^/mm^3^) in blood of male and female mice receiving saline, antibiotics or bacteriophage cocktail. Results are presented as mean values ± SD. Statistical analyses were performed by Kruskal-Wallis test and *post-hoc* Dunn test. The significance of differences between controls and particular treated groups are observed and marked by: asterisks (*) vs. saline control males or saline control females group; (#) vs. bacteriophage males or bacteriophage females group; (&) vs. females; (α) vs. baseline value.
