# Supplementary figures and images for: Sex-dependent differences in behavioral and immunological responses to antibiotic and bacteriophage administration in mice
Source: Front Immunol. 2023 May 25;14:1133358. doi: 10.3389/fimmu.2023.1133358 (PMC10247983; doi:10.3389/fimmu.2023.1133358)

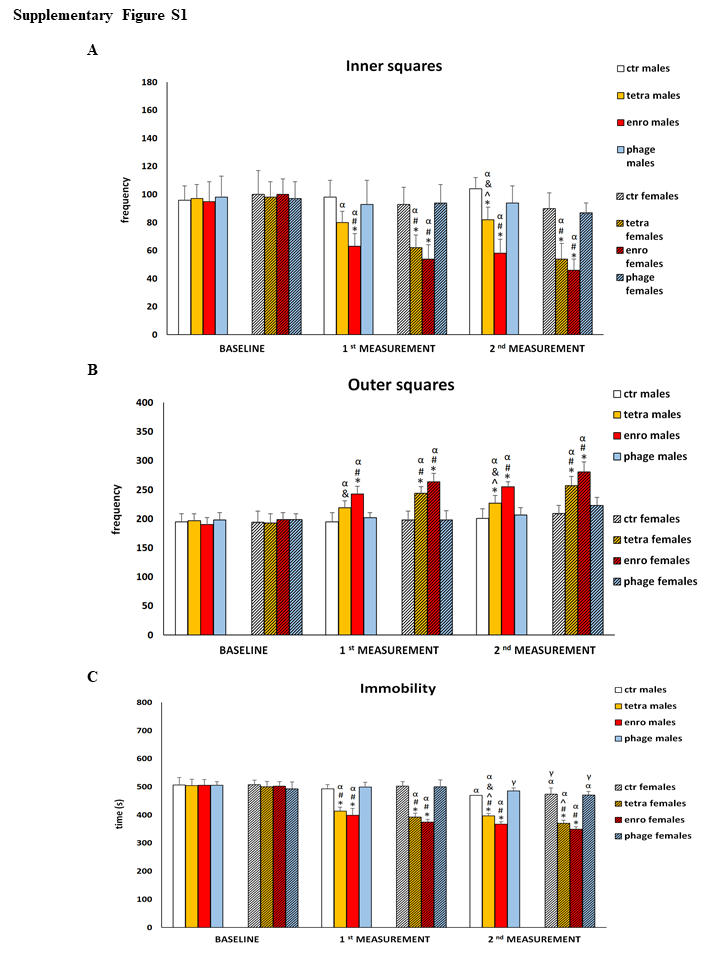

Supplement: Supplementary Figure 1 — Changes in the anxiety behavior in the open field test (10 minutes): (A) frequency of entries to the inner squares; (B) frequency of entries to the outer squares; (C) immobility in male and female mice receiving saline, antibiotics or bacteriophage cocktail. Results are presented as mean values ± SD. Statistical analyses were performed by ANOVA and post-hoc Tukey test. The significance of differences between controls and particular treated groups are observed and marked by: asterisks (*) vs. saline control males or saline control females group; (#) vs. bacteriophage males or bacteriophage females group; (^) vs. enrofloxacin males or enrofloxacin females group; (&) vs. females; (α) vs. baseline value; (γ) vs. 1st measurement value. [file Image_1.tif]

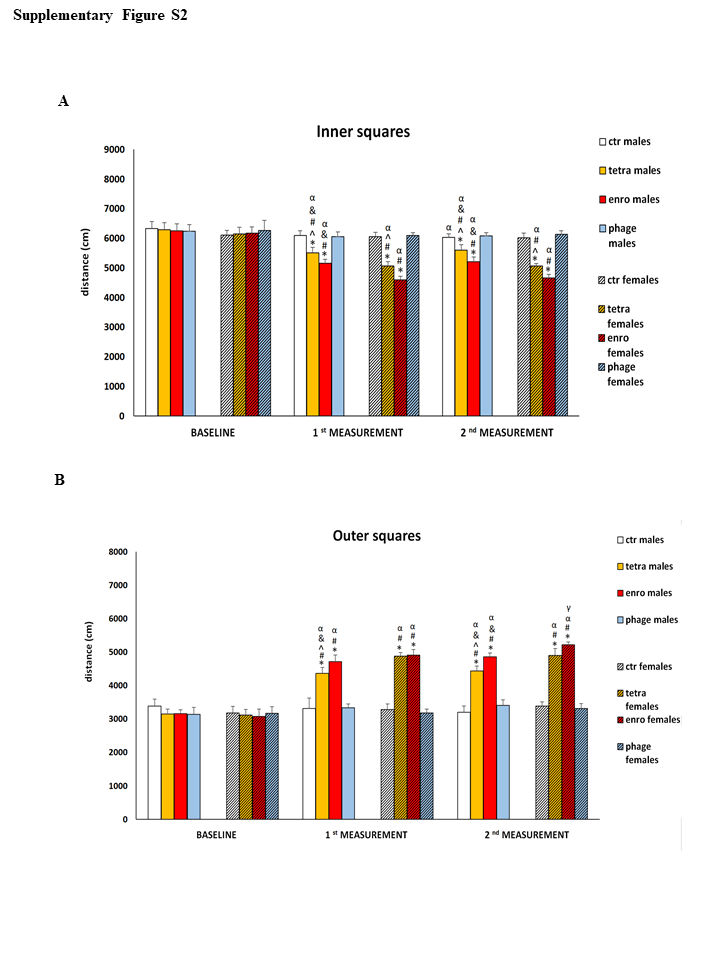

Supplement: Supplementary Figure 2 — Changes in the anxiety behavior in the open field test (10 minutes): (A) distance in the inner squares; (B) distance in the outer squares in male and female mice receiving saline, antibiotics or bacteriophage cocktail. Results are presented as mean values ± SD. Statistical analyses were performed by ANOVA and post-hoc Tukey test. The significance of differences between controls and particular treated groups are observed and marked by: asterisks (*) vs. saline control males or saline control females group; (#) vs. bacteriophage males or bacteriophage females group; (^) vs. enrofloxacin males or enrofloxacin females group; (&) vs. females; (α) vs. baseline value. [file Image_2.tif]

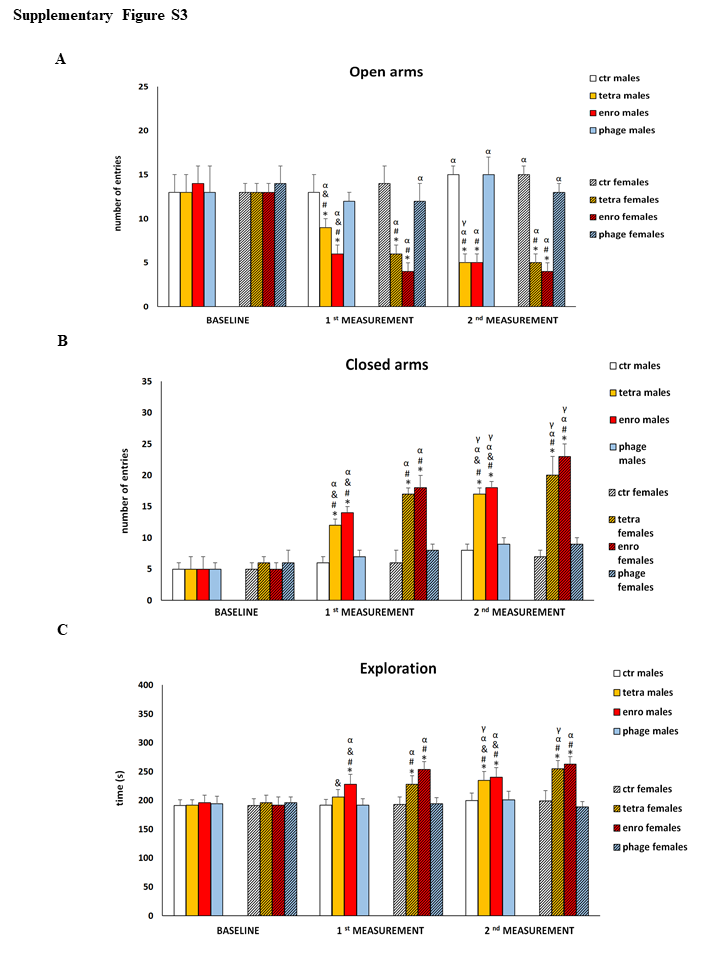

Supplement: Supplementary Figure 3 — Changes in the anxiety behavior and memory processes in the elevated plus-maze test (5 minutes): (A) number of entries to open arms, (B) number of entries to closed arms, (C) exploration in male and female mice receiving saline, antibiotics or bacteriophage cocktail. Results are presented as mean values ± SD. Statistical analyses were performed by ANOVA and post-hoc Tukey test for number of entries to open arms and exploration and by Kruskal-Wallis test and post-hoc Dunn test for number of entries to closed arms. The significance of differences between controls and particular treated groups are observed and marked by: asterisks (*) vs. saline control males or saline control females group; (#) vs. bacteriophage males or bacteriophage females group; (&) vs. females; (α) vs. baseline value; (γ) vs. 1st measurement value. [file Image_3.tif]

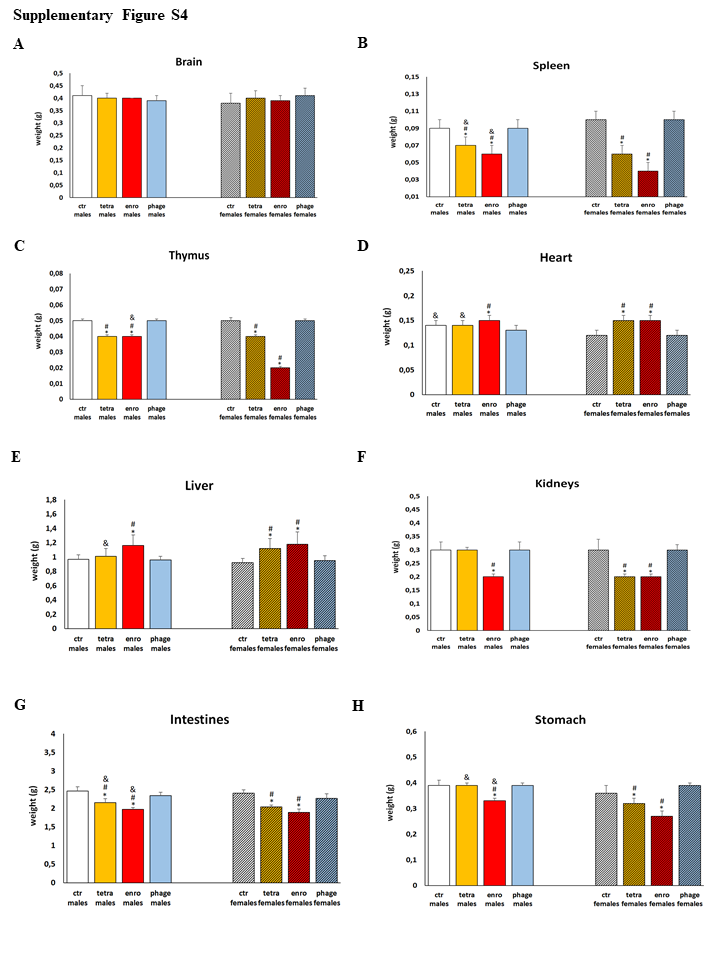

Supplement: Supplementary Figure 4 — Changes in the weights of particular internal organs: (A) brain, (B) spleen, (C) thymus, (D) heart, (E) liver, (F) kidneys, (G) intestines and (H) stomach, after fourteen days of antibiotic or bacteriophage administration in male and female mice. Results are presented as mean values ± SD. Statistical analyses were performed by ANOVA and post-hoc Tukey test. The significance of differences between controls and particular treated groups are observed and marked by: asterisks (*) vs. saline control males or saline control females group; (#) vs. bacteriophage males or bacteriophage females group; (&) vs. females. [file Image_4.tif]

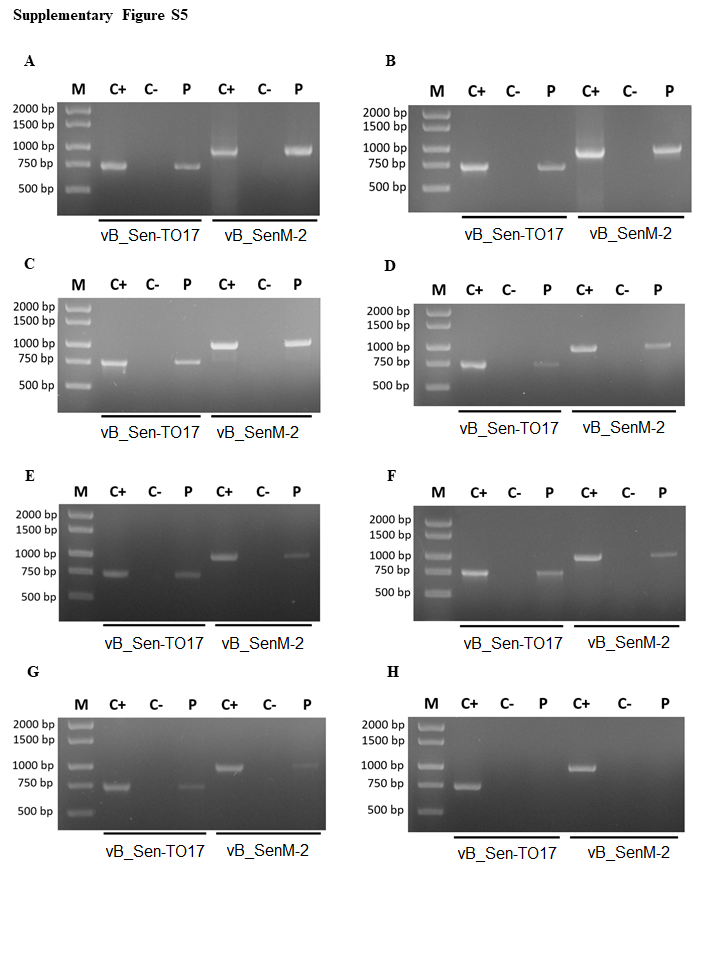

Supplement: Supplementary Figure 5 — Identification of bacteriophages in particular organs: (A) livers of male, (B) livers of female, (C) kidneys of male, (D) kidneys of female, (E) spleens of male, (F) spleens of female, (G) hearts of male, (H) hearts of female mice treated with the bacteriophage cocktail. Specific products of 988 bp (bacteriophage vB_SenM-2) and 736 bp (bacteriophage vB_Sen-TO17) were analyzed by the PCR method. [file Image_5.tif]

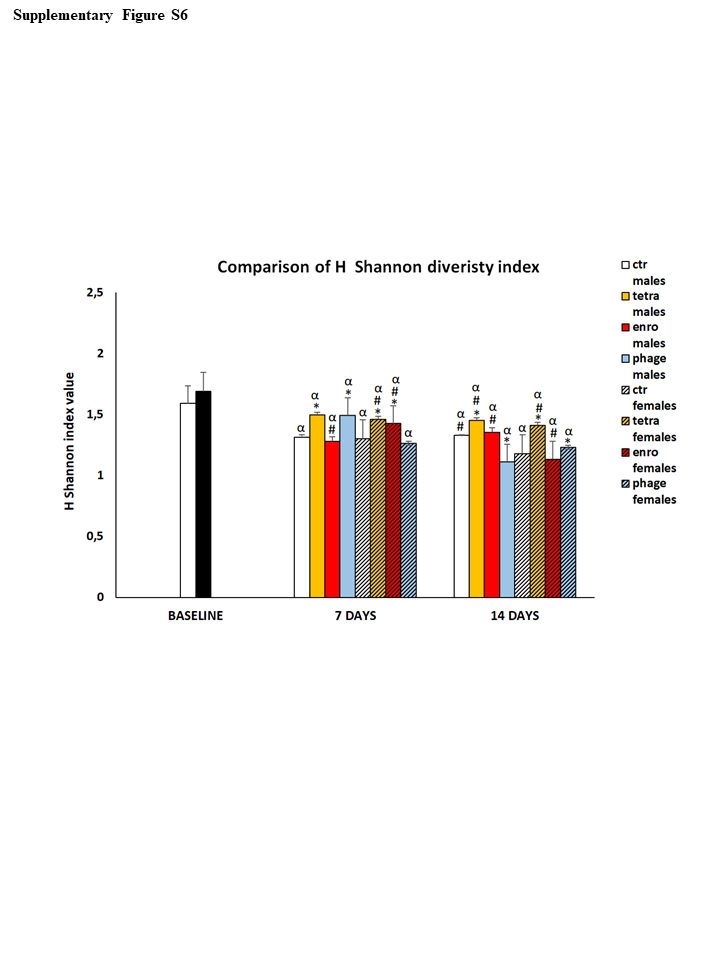

Supplement: Supplementary Figure 6 — The Shannon diversity index, taking into account the OTU (the abundance of each operational taxonomic unit) value. [file Image_6.tif]
