# Supplementary material for: Sex-dependent differences in behavioral and immunological responses to antibiotic and bacteriophage administration in mice
Source: Front Immunol. 2023 May 25;14:1133358. doi: 10.3389/fimmu.2023.1133358 (PMC10247983; doi:10.3389/fimmu.2023.1133358)
Supplement: Supplementary Table 1 — Changes in the absolute (103/µl) counts of leukocytes, lymphocytes, monocytes and granulocytes and relative (%) values of lymphocytes, monocytes and granulocytes in blood of male and female mice receiving saline, antibiotics or bacteriophage cocktail. Results are presented as mean values ± SD. Statistical analyses were performed by Kruskal-Wallis test and post-hoc Dunn test. The significance of differences between controls and particular treated groups are observed and marked by: asterisks (*) vs. saline control males or saline control females group; (#) vs. bacteriophage males or bacteriophage females group; (&) vs. females; (α) vs. baseline value. [file DataSheet_1.pdf]

Supplementary Table S1

Types of cells	Absolute numbers of cells in different groups (No x 10^3/µl)									
		MALES					FEMALES			
	Measurement	EXPERIMENTAL GROUPS				Measurement	EXPERIMENTAL GROUPS			
		CONTROL	TETRACYCLINE	ENROFLOXACINE	PHAGE COCKTAIL		CONTROL	TETRACYCLINE	ENROFLOXACINE	PHAGE COCKTAIL
LEUKOCYTES	BASELINE	7.7±0.5	7.5±0.4	7.4±0.3	7.5±0.6	BASELINE	7.3±0.5	7.4±0.3	7.2±0.3	7.1±0.5
	14 DAYS	7.6±0.5	9.9±0.4 * # & α	11.1±0.6 * # & α	7.9±0.4 &	14 DAYS	7.4±0.6	8.8±0.6 * # α	9.7±0.7 * # α	7.6±0.5
LYMPHOCYTES	BASELINE	6.1±0.5	6.4±0.5	6.2±0.4	6.1±0.6	BASELINE	5.6±0.3	5.8±0.7	6.1±0.6	6.0±0.3
	14 DAYS	6.2±0.7	7.9±0.7 * # & α	9.0±0.4 * # & α	6.4±0.5	14 DAYS	5.9±0.4	7.1±0.6 * # α	8.5±0.3 * # α	6.2±0,3
MONOCYTES	BASELINE	0.3±0.1	0.3±0.1	0.3±0.1	0.2±0.1	BASELINE	0.3±0.1	0.3±0.1	0.3±0.1	0.4±0.1
	14 DAYS	0.3±0,1	0.4±0.1 * # &	0.6±0.1 * # & α	0.3±0.1	14 DAYS	0.4±0.1	0.6±0.1 * # α	0.8±0.1 * # α	0.3±0.1
GRANULOCYTES	BASELINE	0.3±0.002	0.3±0.003	0.,2±0.003	0.3±0.005	BASELINE	0.3±0.002	0.3±0.005	0.2±0.001	0.4±0.004
	14 DAYS	0.3±0.002	0.5±0.005 * # α	0.7±0.005 * # α	0.2±0.003	14 DAYS	0.3±0.004	0.5±0.003 * # α	0.7±0.004 * # α	0.3±0.005
Relative numbers of cells in different groups (%)										
LYMPHOCYTES	BASELINE	77.1±3.1	76.9±2.5	73.5±2.7	75.9±3.4	BASELINE	71.9±3.6	73.4±4.8	74.1±2.5	72.5±3.3
	14 DAYS	76.6±2.8	83.2±4.4 * # & α	89.6±2.5 * # & α	77.4±2.2	14 DAYS	74.6±4.5	79.2±2.5 * # α	85.6±3.7 * # α	74.5±2.6
MONOCYTES	BASELINE	4.6±1.3	4.4±1.5	4.5±1.6	4.3±1.1	BASELINE	4.2±1.5	3.9±1.5	4.5±1.8	4.3±1.7
	14 DAYS	4.4±1.5	8.9±1.9 * # & α	9.8±1.5 * # & α	4.9±1.1	14 DAYS	4.7±1.4	7.5±1.6 * # α	8.7±1.5 * # α	4.5±1.6
GRANULOCYTES	BASELINE	4.1±0.9	3.9±0.7	4.4±0.4	3.9±0.6	BASELINE	4.1±0.8	3.9±0.4	3.8±0.8	4.4±0.6
	14 DAYS	4.0±0.5	7.9±0.8 * # & α	8.9±0.7 * # & α	4.2±0.2	14 DAYS	4.3±0.5	6.6±0.9 * # α	8.1±0.4 * # α	4.3±0.2

Supplementary Table S2

Erythrocyte indices	Measurement	MALES				FEMALES			
		EXPERIMENTAL GROUPS				EXPERIMENTAL GROUPS			
		CONTROL	TETRACYCLINE	ENROFLOXACINE	PHAGE COCKTAIL	CONTROL	TETRACYCLINE	ENROFLOXACINE	PHAGE COCKTAIL
ERYTHROCYTES (No x $10^6/mm^3$)	BASELINE	8.3±0.5 &	8.5±0.8 &	8.7±0.6 &	8.4±0.4 &	7.4±0.3	7.1±0.8	7.5±0.3	7.4±0.8
	14 DAYS	8.2±0.7 &	7.1±0.5 * # & α	6.5±0.6 * # & α	8.2±0.8 &	7.6±0.3	6.5±0.7 * # α	5.9±0.8 * # α	7.6±0.9
HEMOGLOBIN (g/dL)	BASELINE	16.2±0.7	16.9±0.7 &	16.5±0.4	15.9±0.9	15.9±0.3	15.7±0.8	16.0±0.4	15.4±0.5
	14 DAYS	16.7±0.6 &	14.3±0.8 * # & α	12.9±0.5 * # α	16.3±0.8 &	15.7±0.6	13.5±0.7 * # α	12.6±0.7 * # α	15.8±0.9
HEMATOCRIT (%)	BASELINE	48.9±4.2	47.6±5.0	49.7±5.2 &	48.2±2.4	46.8±5.1	45.6±7.1	44.9±8.2	47.1±6.3
	14 DAYS	49.4±6.8	39.8±5.2 * # α	34.8±1.2 * # α	45.6±3.3	45.8±7.1	40.1±6.2 * # α	36.1±1.8 * # α	45.9±2.9
MCV (fL)	BASELINE	49.8±1.4 &	48.4±2.2 &	46.9±1.8 &	47.2±2.3	46.8±2.2	45.9±1.8	43.9±1.9	45.8±1.9
	14 DAYS	50.1±1.4	41.6±2.2 * # α	37.9±2.8 * # α	45.6±3.3	48.2±1.9	38.6±6.2 * # α	34.1±2.5 * # α	46.2±5.3
MCH (pg)	BASELINE	15.4±0.5	15.9±0.7	14.8±0.3	15.4±0.6	14.9±0.6	15.1±0.5	14.2±0.8	14.4±0.8
	14 DAYS	15.6±0.5	13.8±0.6 * # α	12.9±0.6 * # & α	15.8±0.8	14.6±0.8	13.1±0.8 * # α	11.9±0.8 * # α	15.5±0.4
MCHC (g/L)	BASELINE	308.3±9.5	322.8±10.6 &	314.70±9.2 &	326.8±12.9 &	301.4±5.2	304.8±11.6	296.70±7.2	306.8±7.9
	14 DAYS	313.4±6.8 &	283.1±9.6 *# α	244.6±8.6 * # & α	334.2±8.9 & α	299.4±5.8	284.1±7.1 *# α	222.6±4.1 * # α	316.2±1.4 α
PLT (No x $10^3/mm^3$)	BASELINE	741.3±20.8 &	774.5±14.6 * # &	758.8±32.1 * # &	739.9±15.5 &	732.3±9.8	726.5±10.6 #	744.8±22.1 * #	719.9±10.5
	14 DAYS	756.4±22.8 & α	768.5±15.2 * # & α	764.8±30.1 * # & α	742.5±12.1 &	732.4±11.8	743.5±10.2 * # α	751.8±22.1 * # α	732.5±7.1 α
